# Neuroprotection of paeoniflorin as antidepressant candidate

**DOI:** 10.37796/2211-8039.1680

**Published:** 2025-12-01

**Authors:** Yuh-Fung Chen, Yi-Jui Chen, Jai-Sing Yang, Min-Min Lee, Huei-Yann Tsai

**Affiliations:** a Department of Pharmacology, China Medical University, Taichung City 404328, Taiwan, ROC; b Department of Pharmacy, China Medical University Beigang Hospital, Yunlin County 651012, Taiwan, ROC; c Department of Medical Research, China Medical University Hospital, Taichung 404327, Taiwan, ROC; d Department of Food Nutrition and Health Biotechnology, Asia University, Taichung 41354, Taiwan, ROC; e Department of Pharmacy, China Medical University Hospital, Taichung 404327, Taiwan, ROC

**Keywords:** Paeoniflorin, NMDA-mediated EPSP, NMDA-induced calcium influx, Hippocampus, Antidepressant

## Abstract

**Background:**

Depression is one of the common mental disorders worldwide, and currently used antidepressants have undesirable effects; therefore, the development of new antidepressants without side effects is urgently needed. Paeoniflorin (PF) exhibits various pharmacological activities, including anti-inflammatory, antioxidant, and neuroprotective effects. NMDA receptors in the hippocampus play a vital role in the pathophysiology of depression. Due to the scarcity of reports on the neuroprotection of PF on NMDA-induced excitotoxicity in the hippocampus, the present study aims to investigate the effects of PF on NMDA-mediated EPSP and calcium influx in the hippocampus to evaluate the potential of PF as an antidepressant.

**Methods:**

In order to investigate the effects of PF on the NMDA receptor in the hippocampus, the hippocampal slices, primary-cultured hippocampal neurons, and *in silico* molecular docking analysis of PF with the NMDA receptor were used.

**Results:**

PF (2 μM) significantly depressed the NMDA-mediated EPSPs, resulting in a 50 % inhibition. The intracellular calcium level in primary-cultured hippocampal neurons was 102.67 nM, and 520.36 nM after NMDA (125 μM) treatment. With NMDA and PF co-treatment, the calcium level was 204.58 μM, showing a 60.68 % decrease. After NMDA was co-treated with 1 μM ruthenium red (RuR), the calcium level increased (from 534.58 nM to 665.68 nM). Additionally, co-treatment with PF significantly decreased the calcium level (468.05 nM, representing a 29.50 % decrease). In the presence of NMDA and 1 μM ω-conotoxin MVIIC (ω-Cono) co-treatment, the calcium level was 496.29 nM. In the presence of NMDA, ω-Cono, and RuR, the calcium level was 568.5 nM. Additionally, NMDA, ω-Cono, RuR, and PF co-treatment significantly decreased the calcium level to 270.94 nM. In silico molecular docking analysis revealed a binding energy of −48.5188 kcal/mol for PF with the NMDA receptor.

**Conclusions:**

PF binds to the NMDA receptor, exhibits neuroprotection, and contributes to its potential as an antidepressant.

## 1. Introduction

Depression is one of the common mental disorders worldwide, with its prevalence continuing to increase and it has become a global health burden [1]; it is a multifactorial disease involving several interrelated pathways, and neuroinflammation is one of the vital pathways. Neuroinflammation, which promotes oxidative stress, leads to neuronal damage. Glutamate-induced excitotoxicity is involved in the onset and progression of depression [2,3]. Currently used antidepressants include tricyclic antidepressants (TCAs), monoamine oxidase inhibitors (MAOIs), and selective serotonin reuptake inhibitors (SSRIs). However, these antidepressants have serious side effects, such as drowsiness, dry mouth, headache, nausea, and sexual dysfunction [4,5]. The development of new drugs for depression without the above side effects is urgently needed.

The hippocampus is a crucial brain region in regulating the stress response [6], and hippocampal neuron loss resulting from stress exposure has been extensively studied in animal models [7–11]. The glutamatergic system in the hippocampus is also involved in the pathophysiology of depression, as has been reported [12,13]. Glutamate is released and binds to its receptor upon stimulation [14]. N-methyl-d-aspartate receptors (NMDARs), one of the glutamate receptor subtypes, mediate excitatory neurotransmission by allowing Ca^2+^ to pass through the channel and play an important role in synaptic plasticity and memory function [15]. Several studies provide evidence for a role of hippocampal NMDARs in anxiety and depression [10,11,16]. Any disruption in this pathway may lead to a change in its activity and be attributed to the dysfunction of NMDARs, which results in neuropsychiatric pathologies [17]. In the 1990s, the NMDARs were first proposed to be involved in the pathophysiology of depression, and MK801 (dizolcipine) was found to mimic antidepressant-like effects in animal behavior studies, such as the forced swimming test and tail-suspension test [18,19]. Ketamine has been used as a rapid-acting antidepressant that reinforced the involvement of NMDA receptors in depression [20]. Therefore, the role of NMDAR in the pathophysiology and therapy of mental disorders has been widely discussed [21]. Previous studies indicated that NMDA receptor antagonists exert their anxiolytic effects by blocking the NMDA receptor in the ventral hippocampus [11], revealing that glutamate/NMDA receptor signaling plays a crucial role in alleviating mood disorder symptoms and serves as a therapeutic target for antidepressants [15,22–24].

PF is one of the active components extracted from *Paeonia lactiflora* Pallas and exhibits a wide range of pharmacological activities, including anti-inflammatory [25], antioxidant [26], anticonvulsant [27], analgesic [28], hepatoprotective [29], and antidepressant effects [30], as well as the ability to attenuate cerebral ischemia and arterial intimal hyperplasia [31]. Previous reports indicate that PF can regulate key mediators associated with the pathophysiology of depression, including BDNF, CREB [32], NF-κB, TLR-4 [33], NLRP3 [34], the HPA axis, serotonin [35], mTOR [36], HMGB1 [37], ROS, caspases [38], and SNARE proteins [39], thereby exerting its antidepressant effects in animal studies. PF treatment significantly reduced hippocampal glutamate levels compared with the prenatally stressed model group [40]. Due to the importance of glutamate/NMDAR signaling in alleviating symptoms of mood disorders, and the scarcity of reports on the interaction between PF and NMDA-induced excitotoxicity, the present experiment aims to investigate the effects of PF on NMDA-mediated EPSP and calcium influx in the hippocampus. The hippocampal slices, primary cultured hippocampal neurons, and *in silico* molecular docking analysis of PF with NMDA receptor complex will be used in the present study.

## 2. Materials and methods

### 2.1. Chemicals and reagents

The chemicals were purchased from the following companies. (±)-α-amino-3-hydroxy-5-methyl-isoxazole-4-propionic acid (AMPA), boric acid, calcium chloride (CaCl_2_·2H_2_O), Cytarabin (Ara-C), deoxyribonuclease I (DNase I), dimethyl sulphoxide (DMSO), ethylenediaminetetraacetic acid (EDTA), monoclonal anti-glial fibrillary acidic protein (GFAP 1:500), glutamate, l-cysteine, N-(2-hydroxyethyl) piperazine-N′-(2-ethanesulfonic) (HEPES), magnesium sulfate (MgSO4), N-methyl-d-aspartic acid (NMDA), papain, pluronic F-127, poly-d-lysine, picrotoxin, potassium chloride (KCl), pyruvic acid, ruthenium red, sodium chloride, sodium bicarbonate (NaHCO_3_), sodium dihydrogen phosphate (NaH_2_PO_4_) and verapamil were Sigma–Aldrich (St. Louis, MO, USA). (+)-MK-801 hydrogen maleate was from TCI, Japan. B-27, fetal bovine serum (FBS), glutamine, horse serum, phosphate buffered saline (PBS), penicillin/streptomycin (P/S), picrotoxin and trypan blue stain 0.4 % were from Thermo Fisher Scientific. Microtubule associated protein-2 (MAP-2 1:500) was from Bochringer Mannheim, Germany. 2,3-Dioxo-6-nitro-1,2,3,4-tetrahydrobenzo [f] quinoxaline-7-sulfonamide (NBQX) and ω-conotoxin MVIIC were from TORIS, England. Paeoniflorin was from Yoneyama Yakuhin Kogyo CO., LTD, Japan. Fura-2, fura-2/AM (fura-2 acetoxymethyl), Dulbecco’s Modified Eagle Medium (DMEM), neurobasal medium, and Hank’s balanced salt solution (HBSS) were obtained from ThermoFisher Scientific (Waltham, MA, USA).

### 2.2. Preparation of hippocampal slices and primary cultured hippocampal neurons

1-month-old Sprague–Dawley (SD) rats, weighing approximately 75–100 g, were used for the preparation of hippocampal slices, and primary cultured hippocampal neurons were prepared using postnatal 0-day-old rat brains. SD rats were purchased from the National Laboratory Animal Center, Taipei, Taiwan. All animals were fed with standard chow and housed at a constant temperature (22 ± 1 °C) with relative humidity (55 ± 5 %) and with a 12 h inverted light–dark cycle for one week before the experiments. Surgery was performed under Zoletil® anesthesia. The preparation protocols of hippocampal slices [41] and primary-cultured hippocampal neurons were modified as previously described [42]. All animal procedures were approved by the Institutional Animal Care and Use Committee (IACUC) of China Medical University, which reviewed and approved this animal experimental protocol (CMU-IACUC102-224).

#### 2.2.1. Effect of PF on NMDA receptor-mediated excitatory postsynaptic potentials (EPSPs)

In the central nervous system, glutamate is an important excitatory neurotransmitter released by excitatory synapses, and the NMDA receptor is its primary receptor. Therefore, this series of experiments was designed to investigate the effects of PF on NMDA receptor-mediated excitatory postsynaptic potentials (field-EPSPs). Electrophysiological techniques were employed further to investigate the effects of PF on NMDA receptors.

The preparation of hippocampal slices was used for eliciting CA1 field-EPSPs as previously described [41]. 1-month-old rats, weighing approximately 75–100 g, were decapitated, and the hippocampus was rapidly removed. Using a vibrating microtome, the hippocampus is sliced perpendicular to its long axis into brain slices of 400–450 μm thickness. The slices were maintained in ice-cold artificial cerebrospinal fluid (ASCF) containing in mM: 119 NaCl, 12.5 KCl, 2.5 CaCl_2_, 1.3 MgSO_4_, 1 NaH_2_PO_4_, 26.2 NaHCO_3_, and 11 glucose at pH 7.4 and equilibrated with 95 % O_2_ and 5 % CO_2_. Hippocampal slices were placed in an interface slice chamber at room temperature for 1.5–2 h until neuronal activity recovered. The hippocampal slices were then transferred to a submersion-type slice chamber and perfused with ASCF at a stable flow rate at room temperature for experimental recording. At this point, 0.1 mM picrotoxin was added to ACSF to block GABA_A_ receptor-mediated inhibitory synaptic activity. This experiment focuses on the synapses where the Schaffer collateral branches of CA3 pyramidal cells project onto CA1 pyramidal cells. To prevent GABA_A_ receptor blockade caused by epileptic form firing generated by CA3 pyramidal cells, the connection between CA1 and CA3 in the hippocampal slice was severed to interrupt neural transmission. Place a glass recording electrode filled with 3 M NaCl (resistance 4–6 MΩ) in the stratum radiatum layer of the CA1 region to record extracellular EPSP signals (field-EPSP). A bipolar stimulus was placed in the stratum radiatum layer near the recording electrode to stimulate the Schaffer collateral branch. The stimulation frequency was set to 0.1 Hz. The stimulation intensity was adjusted to obtain a stable field-EPSP without field spikes (cell action potentials), for the induction of NMDA receptor-mediated field-EPSPs, AMPA receptor antagonist DNQX 10 μM was used to block AMPA receptors, followed by perfusion with ACSF containing DNQX 10 μM and 0.1 mM Mg^2+^ to highlight the action of NMDA. After obtaining stable NMDA receptor-mediated field-EPSPs, the stimulus intensity was fixed, and field-EPSPs were elicited at the same stimulus frequency (0.1 Hz) and recorded for 10 min. PF was then added to the organ bath, and recordings were made for 10–15 min before PF was washed away. Data were acquired by digitizing signals using a CED 1401 (Cambridge Electronic Design) interface, then displayed online using CED signal software and stored on a PC. Field-EPSP is measured by quantifying the slope of the initial segment of EPSP. Four different concentrations of PF were used in perfusion to determine the ID_50_, which was then used in subsequent experiments.

#### 2.2.2. Preparation of primary cultured hippocampal neurons

The preparation of primary cultured hippocampal neurons was modified as previously described [42]. Pregnant SD female rats were purchased from the National Laboratory Animal Center, Taipei, Taiwan. After delivery, pups were removed within 12 h of birth and anesthetized by intraperitoneal injection of 0.1 mL Zoletil®. The head and whole body were wiped with a 70 % alcohol swab, then the whole brain was rapidly removed from the cranium by cutting the skull using a curved scissor and placed in a 35 mm Petri dish containing ice-cold incomplete medium (100 mL DMEM + 0.5 mL). Under a dissecting microscope, blood vessels, meninges, and other tissues were carefully removed, and the hippocampus was separated and placed into a centrifuge tube containing ice-cold incomplete medium. 1 mL of plastic pipette was used to titrate the removed hippocampus 30~50 times to disperse the large pieces of tissue thoroughly and homogenize, and 5 mL of papain solution was added. After shaking at 125 rpm for approximately 20 min, 1 mL of horse serum was added to terminate the reaction of the papain solution. Centrifuged the cell suspension at 200×*g* for 2 min, removed the supernatant, then added 5 mL incomplete medium, and then aspirated with a 10 mL plastic pipette 50 times to form a cell suspension. The cell suspension was centrifuged at 200×*g* for 2 min, and the supernatant was removed. The cell suspension was then filtered through a 70 μm nylon cell strainer (FALCON®) and collected. Added 5 mL of incomplete medium and 1.1 mL of horse serum to the cell suspension, then titrated the filtered hippocampal cells well. Took 100 μl of hippocampal cell suspension, and 100 μl of TrypBlue dye was added to mix well and stand for 1 min. 100 μl of the cell mixture was injected into a cytometer (Hemocytometer) and counted under a microscope. A suspension of 10^3^ hippocampal cells was quantitatively planted in 25 mm coverslips containing poly-D-Lysin, and the cells were allowed to form a monolayer on the coverslip. After 2 h, the medium was changed to a new medium (neurobasal medium containing 10 % B-27, 1 % glutamate, and 1 % glutamine). This procedure was followed by a further change to a new medium (neurobasal medium containing 10 % B-27 without glutamate and glutamine) for 4 days. Then they were changed to fresh medium every two days, and the primary cultured hippocampal neuronal cells were cultured for 10~14 days for the Ca^2+^ analysis.

#### 2.2.3. Hippocampus section identification

The previously removed hippocampal tissue was placed in 10 % formalin for fixation for 24 h and then soaked in 70 %, 80 %, 95 % (twice), and 100 % (four times) alcohol for 1 h each, then embedded the hippocampal tissue in paraffin, and then sliced the tissue in a rotary microtome. Sections were stained with hematoxylin/eosin (H&E) and examined under a microscope for hippocampus identification.

#### 2.2.4. Immunocytochemistry

After removing the culture medium from the primary-cultured hippocampal neuronal cells on the 4^th^, 10^th^, and 14^th^ day of culture, the cells were washed three times with PBS (phosphate-buffered saline) for 5 min each time. The primary-cultured hippocampal neuronal cells were immersed in and fixed with 4 % (v/v) paraformaldehyde for 10 min. They were then rinsed five times with PBS and treated with 3 % H_2_O_2_ for 10 min. Subsequently, TBST (0.5 mL Tween 20 in 1000 mL Tris-buffered saline) was added. After 10 min of H_2_O_2_ immersion, the cells were washed twice in TBST for 5 min each time. Then the cells were reacted with a non-specific blocking agent (5 % milk in TBST) for 30 min at room temperature to prevent non-specific binding. After that, TBST was soaked twice for 5 min each time (control group). In the experimental group, MAP-2 antibody (1:500) was added. The reaction was carried out at room temperature for 90 min, and in the neuroglial cell group, GFAP antibody (1:500) was added. The reaction was carried out at room temperature for 90 min, and each group was washed three times with TBST for 5 min each time. GFAP antibody (1:500) was added to the neural collagen cell group, and the reaction was performed at room temperature for 90 min. The cells were then washed three times with TBST for 5 min each time. HRP was added to stop the collagen reaction for 20 min. The mixture was then washed with TBST three times, each time for 5 min. Then the reaction was carried out in a DAB solution (DAB 0.05g, 30 % H_2_O_2_ 67 μl in 100 mL 1× PBS buffer) was used to present the color for 10 min, and then the reaction was washed three times with TBST for 5 min each time to stop the reaction. Nuclei were stained with Hematoxylin and dehydrated with 70 %, 85 %, 95 % (twice), and 100 % (twice) alcohol for 20–30 s each time, and then dried. The Image Plus imaging system was used to analyze the results of section staining.

### 2.3. Determination of the NMDA-induced calcium influx in primary-cultured hippocampal neurons

Hippocampal primary-cultured neuronal cells were first placed in 2 μM Fura-2/AM and incubated at room temperature for 30 min. Then, the old medium was washed with HEPES buffer, and 1 mL of fresh DMEM was added. The coverslip dish was then placed under a fluorescent microscope, and the signal was detected at 510 nm to observe the changes in Ca^2+^ levels in the primary-cultured hippocampal neuronal cells.

The simultaneous excitation of Fura-2-Ca^2+^ measured changes in Ca^2+^ levels in primary-cultured hippocampal neuronal cells, and the excitation signals were intercepted by a cool CCD, and analyzed by a computer using MetaFlour® Imaging software (Universal Imaging Corporation, Chester, PA), using fluorescence ratios (R = 340/380 nm), which was calculated as follows: [Ca^2+^]*_i_* = Kd(F_0_/F_s_) [(R−R_min_)/(R_max_−R)]

Primary-cultured hippocampal neurons were treated with 125 μM NMDA for 45 min, then incubated with 2 μM Fura-2/AM, and intracellular calcium levels were measured using the method as previously described.

### 2.4. Effect of different concentrations of PF on NMDA-induced calcium influx in primary cultured hippocampal neurons

Different concentrations of PF (1–30 μM) and 125 μM NMDA were added to the primary-cultured hippocampal neurons for 45 min, and 2 μM Fura-2 AM was added to the treated primary-cultured hippocampal neurons for 30 min. Intracellular calcium levels were measured for 5 min using the method as previously described. Besides, 270 s after 125 μM NMDA-treated primary-cultured hippocampal neurons, PF (10, 30, 90 μM) was added to the primary-cultured hippocampal neurons, respectively. Additionally, repeated application of PF (10 or 30 μM) to the NMDA-treated primary-cultured hippocampal neurons was also performed to evaluate the effects of repeated PF application on the changes of calcium levels in the NMDA-treated primary-cultured hippocampal neurons. The intracellular calcium levels were measured for 30–45 min.

### 2.5. Effects of calcium channel modulators and PF on the NMDA-induced calcium influx in primary-cultured hippocampal neurons

#### 2.5.1. Effects of ruthenium red (RuR) co-administered with PF on the NMDA-induced calcium level in primary cultured hippocampal neurons

RuR is a non-competitive inhibitor of the mitochondrial Ca^2+^ uniporter, which abolishes Ca^2+^ uptake [43]. Add 1 μM RuR and/or 10 μM PF to the 125 μM NMDA-treated primary-cultured hippocampal neurons for 45 min, then add 2 μM Fura-2 AM to the treated primary-cultured hippocampal neurons and incubate at 37 °C for 30 min. Then, they were washed with HEPES buffer to remove the old medium and observed under a fluorescence microscope using 340 nm and 380 nm wavelengths to excite Fura-2-Ca^2+^, with signals scattered at 510 nm, to detect changes in Ca^2+^ levels in the primary-cultured hippocampal neurons for 10 min.

#### 2.5.2. Effects of ω-conotoxin MVIIC (ω-Cono) on NMDA-induced calcium influx and neuron morphology in primary-cultured hippocampal neurons

ω-Cono is a peptide toxin that inhibits N-type calcium channels [44]. After adding 125 μM NMDA and 1 μM ω-Cono to primary-cultured hippocampal neurons, add 10 μM PF with/or without 1 μM RuR for 45 min. Then add 2 μM Fura-2 AM to the treated primary-cultured hippocampal neurons and incubate at 37 °C for 30 min. The changes in intracellular calcium concentration were measured over a 10-min period.

#### 2.5.3. Effects of ω-conotoxin MVIIC (ω-Cono) or combined with PF and/or with RuR on NMDA-induced calcium influx and neuron morphology in primary-cultured hippocampal neurons

After adding 125 μM NMDA, 1 μM ω-Cono coadministered with 10 μM PF, or additionally in the presence of 1 μM RuR, co-administered with 10 μM PF to primary-cultured hippocampal neurons for 45 min, the changes in intracellular calcium levels were measured for 10 min.

### 2.6. In silico molecular docking analysis of the PF with NMDA receptor complex

The 3D crystal structure of the NMDA receptor protein was obtained from the Protein Data Bank (http://www.rcsb.org/pdb) using the PDB code: 7EOT. Discovery Studio 2022 software (BIOVIA, San Diego, CA, USA) was used to dock PF and GluN1/GluN2A NMDA receptor and visualize the docking results [45,46]. The dock score of CDOCKER interaction energy was used to estimate the binding energy of the ligands with receptor. On the docked conformations, the pose with the highest value of CDOCKER interaction energy score was selected for the calculation of binding energy.

### 2.7. Statistical analysis

All data were expressed as mean ± SE. Single variable comparisons used the Student’s *t*-test, and for variable comparisons used the one-way ANOVA followed by Dunnett’s test. When a P value < 0.05 was regarded as statistically significant.

## 3. Results

### 3.1. Effect of PF on NMDA receptor-mediated excitatory postsynaptic potentials (EPSPs)

**Fig. 1:**
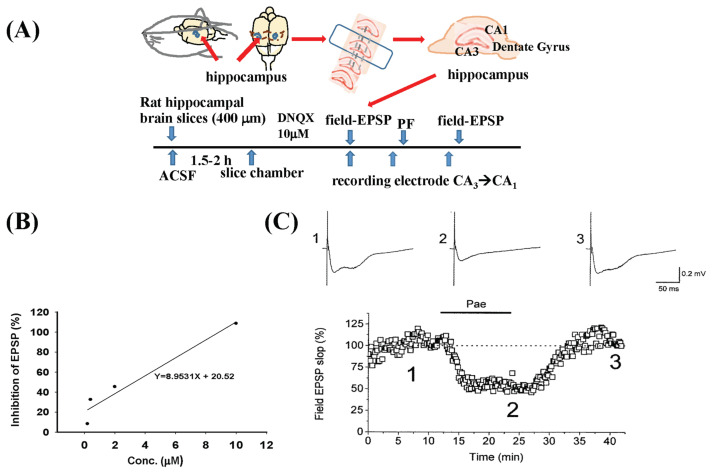
Effect of PF on NMDA receptor-mediated excitatory postsynaptic potentials (EPSPs). The procedure flow chart is shown in Fig. 1(A). The linear regression for different doses of PF is shown in 1B. After performing the linear regression, the ID_50_ (50 % inhibition of EPSP) was calculated to be 3.3 μM. The effect of 2 μM PF significantly reduced NMDA-mediated EPSPs (50 % inhibition) in rat hippocampal brain slices, as shown in 1C.

**Fig. 2:**
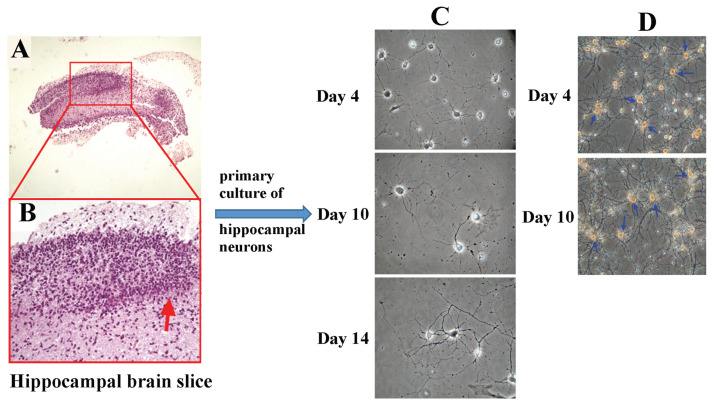
Identification of hippocampal slice and primary-cultured hippocampal neurons by immunocytochemistry was observed. The brain slice obtained from a newborn rat, stained with H&E, was observed under a microscope at 40× magnification (Fig. 2A) and 200× magnification (Fig. 2B). The curved region indicated by the red arrow showed deep red comb-like arrangements of neurons, confirming the hippocampus (Fig. 2B). The primary cultured neurons observed under an inverted microscope on days 4, 10, and 14 after seeding of hippocampal cells, as shown in Fig. 2C. The primary cultured hippocampal neurons were stained with immunocytochemistry on day 4 and day 10 (Fig. 22D). The blue arrow points to a neuronal cell, whose axons and dendrites had not yet formed a high-density network on day 4 (upper panel of Fig. 2D). By day 10 (lower panel of Fig. 2D), the axons and dendrites had formed a distinct high-density network.

**Fig. 3:**
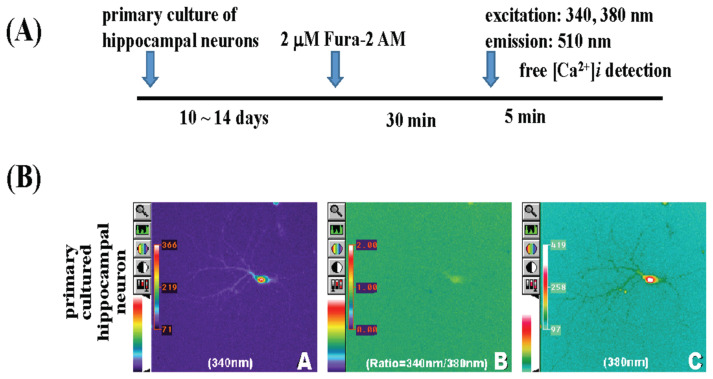
Determination of calcium level in primary-cultured hippocampal neurons. The procedure flow chart is shown in Fig. 3(A). Images were captured using MetaFlour® Imaging, as shown in Fig. 3B (A–C). In Fig. 3B(A), the fluorescence image of primary-cultured hippocampal neurons is shown at 340 nm (200×) and at 380 nm (200×), as shown in Fig. 3(C); Fig. 3(B) displays the fluorescence ratio (R = 340/380 nm) image (200×).

As shown in Fig. 1B, the linear regression for different doses of PF was depicted. The doses of PF used were 0.2, 0.4, 2, and 10 μM. After performing the linear regression, the ID_50_ (50 % inhibition of EPSP) was calculated to be 3.3 μM. The effect of PF 2 μM on NMDA receptor-mediated EPSPs in rat hippocampal brain slices was recorded, and PF (2 μM) significantly depressed the NMDA-mediated EPSPs, resulting in a 50 % inhibition as shown in Fig. 1C. Fig. 1C-1 showed NMDA-mediated EPSPs before PF administration. Fig. 1C-2 showed that the NMDA-mediated potential was significantly reduced during the application of PF (50 % inhibition). Fig. 1C-3 showed the potential response after PF was washed away, with the potential returning to initial levels.

### 3.2. Identification of hippocampal slice and primary-cultured hippocampal neurons by immunocytochemistry

As shown in Fig. 2A, this section was obtained from the hippocampus of a newborn rat, fixed, dehydrated, sectioned, and stained with hematoxylin/eosin (H&E), then observed under a microscope (40×). At 200× magnification, the curved region indicated by the red arrow showed distinct deep red comb-like arrangements of neurons, confirming that the extracted brain tissue was from the hippocampus (Fig. 2B).

Primary-cultured neurons were observed under an inverted microscope on days 4, 10, and 14 after implantation of hippocampal cells (Fig. 2C). The primary-cultured hippocampal neurons were stained with immunocytochemistry staining on day 4 and day 10 (Fig. 2D). The blue arrow points to a neuronal cell, whose axons and dendrites had not yet formed a high-density network on day 4 (upper panel of Fig. 2D), and on day 10 (lower panel of Fig. 2D) showed that the axons and dendrites formed a distinct high-density network. In immunocytochemical staining of primary-cultured hippocampal neurons, the differences between neurons and glial cells were visible. As shown in Fig. 2D, the morphology of hippocampal neurons appeared dendritic under fluorescence microscopy, and the results of immunocytochemical staining revealed that the percentage of neurons among all cells accounts for approximately 85.4 % (as shown in Fig. 2D).

### 3.3. Determination of calcium level in primary-cultured hippocampal neurons

Primary-cultured hippocampal neurons were incubated in 2 μM Fura-2/AM at 37 °C for 30 min, then transferred to a fluorescence microscope to detect Fura-2-Ca^2+^ excitation at 340 nm and 380 nm wavelengths, with signals scattered at 510 nm as shown in the protocol in Fig. 3A. Images were captured simultaneously by a computer using MetaFlour® Imaging as shown in Fig. 3B (A~C): (A) shows the fluorescence image of primary-cultured hippocampal neurons at 340 nm (200×); (C) shows the fluorescence image of primary-cultured hippocampal neurons at 380 nm (200×); (B) shows the fluorescence ratio (R = 340/380 nm) image (200×). The Ca^2+^ level in primary-cultured hippocampal neurons at the resting state was 102.67 ± 21.6 nM as shown in Fig. 6.

### 3.4. The effect of NMDA on intracellular Ca^2+^ level and neuron morphology in primary-cultured hippocampal neurons

**Fig. 4:**
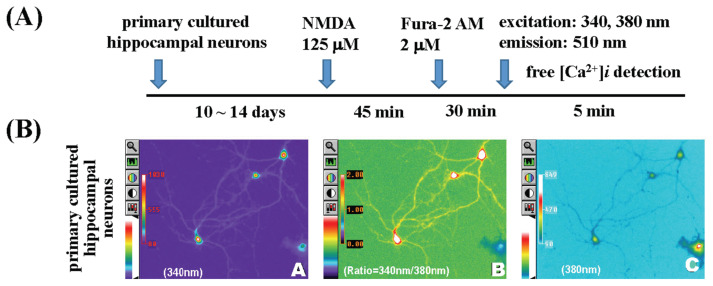
The effect of NMDA on intracellular Ca2+ level and neuron morphology in primary-cultured hippocampal neurons. The procedure flow chart is shown in Fig. 4(A). Imaging as shown in the lower panel of Fig. 4B (A~C): Fig. 4B(A) showed the fluorescence image of primary-cultured hippocampal neurons stimulated by NMDA at 340 nm (200 × magnification); Fig. 4B(C) is an image of primary-cultured hippocampal neurons stimulated by NMDA at 380 nm fluorescence (200×); Fig. 4B(B) is an image of the changes in fluorescence ratio (R = 340/380 nm) (200×). The fluorescence is significantly increased in NMDA-treated primary-cultured hippocampal neurons.

Primary-cultured hippocampal neurons were incubated with 125 μM NMDA for 45 min, and then 2 μM Fura-2/AM was added. The cells were then cultured at 37 °C for 30 min, as shown in the protocol in Fig. 4A. Imaging as shown in the lower panel of Fig. 4B (A~C): 4B(A) showed the fluorescence image of primary-cultured hippocampal neurons stimulated by NMDA at 340 nm (200× magnification); 4B(C) is an image of primary-cultured hippocampal neurons stimulated by 125 μM NMDA at 380 nm fluorescence (200×); 4B(B) is an image of the changes in fluorescence ratio (R = 340/380 nm) (200×).

**Fig. 5:**
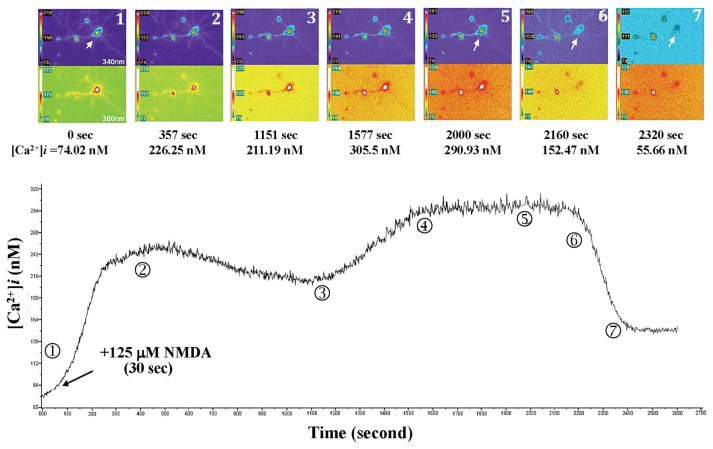
Added NMDA to the primary-cultured hippocampal neurons 30 s after the beginning of fluorescent exposure, which affected the intracellular Ca^2+^ level and neuron morphology. When NMDA was added to the primary-cultured hippocampal neurons at 30 s after the beginning of fluorescent exposure, the intracellular calcium level increased from 74.02 nM (0 s) to 305.5 nM (1577 s). The changes in neuron morphology are shown in the upper panel. The time-course changes in Ca^2+^ level in primary-cultured hippocampal neurons after NMDA challenge are shown in the lower panel. The morphology of primary-cultured hippocampal neurons underwent significant axonal changes at 1151 s. Moreover, as indicated by the arrow at 2000 s, the indicated neuron ruptured (at 2160 s) and died (at 2320 s).

**Fig. 6:**
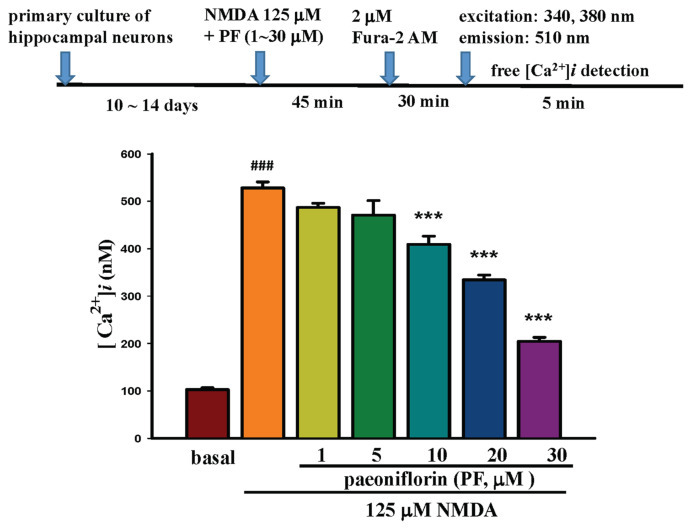
Effects of different concentrations of PF on NMDA-induced calcium influx and neuron morphology in primary cultured hippocampal neurons. The procedure flow chart is shown in upper panel of Fig. 6. PF (10–30 μM) significantly (P < 0.001) decreased NMDA-induced Ca^2+^ influx in primary-cultured hippocampal neurons, as shown in the lower panel. The percentage decrease caused by PF on NMDA-induced intracellular calcium increase was 21.22 %, 35.75 %, and 60.68 %, respectively.

When 125 μM NMDA was added to the primary-cultured hippocampal neurons at 30 s after the beginning of fluorescent exposure. The neurons were exposed for 45 min, the changes of intracellular calcium level were observed, as shown in Fig. 5. The intracellular calcium level was from 74.02 nM (0 s) to 305.5 nM (1577 s), and the changes in neuron morphology as shown in the upper panel of Fig. 5. The time-course changes in Ca^2+^ levels in primary-cultured hippocampal neurons after NMDA challenge are shown in the lower panel of Fig. 5. The morphology of primary-cultured hippocampal neurons showed significant axonal changes at 1151 s. Moreover, as indicated by the arrow at 2000 s, the indicated neuron ruptured (at 2160 s) and died (at 2320 s). After incubation with 125 μM NMDA, the intracellular Ca^2+^ level in primary-cultured hippocampal neurons was 534.58 ± 91.97 nM, as shown in Fig. 6.

### 3.5. Effects of different concentrations of PF on NMDA-induced calcium influx and neuron morphology in primary cultured hippocampal neurons

Primary-cultured hippocampal neurons were incubated with 125 μM NMDA and different concentrations of PF (1–30 μM) for 45 min, then incubated with 2 μM Fura-2/AM for 30 min. Changes in intracellular Ca^2+^ levels in primary-cultured hippocampal neurons were observed using the method described in the text. Different concentrations of PF (10–30 μM) significantly (P < 0.001) decreased 125 μM NMDA-induced Ca^2+^ influx in primary-cultured hippocampal neurons, as shown in the lower panel of Fig. 6. The decrease percentage of PF (10–30 μM) on 125 μM NMDA-induced intracellular calcium increase was 21.22 %, 35.75 %, and 60.68 %, respectively.

**Fig. 7:**
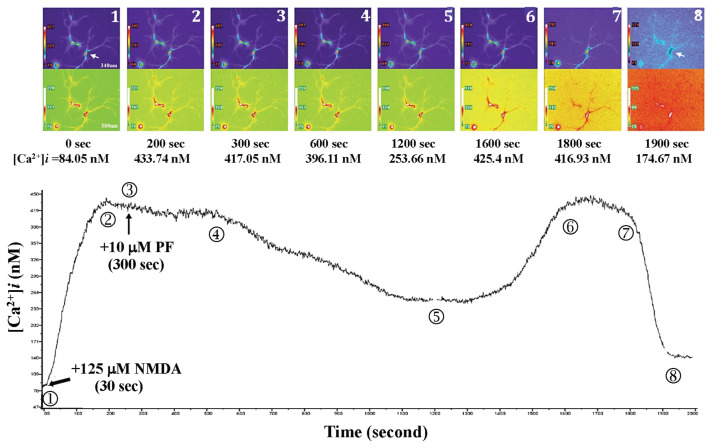
Effects of 10 μM PF on NMDA-induced calcium influx and neuronal morphology in primary cultured hippocampal neurons. When NMDA was added to the primary-cultured hippocampal neurons at 30 s after the beginning of fluorescent exposure, the Ca^2+^ level rose from 84.05 nM (0 s) to 433.74 nM (200 s). PF was added at 300 s, and the Ca^2+^ level decreased to 417.05 nM. Normal morphology was observed in primary-cultured hippocampal neurons. After prolonged fluorescence exposure (at 1200 s), the intracellular Ca^2+^ level began to rise (253.66 nM at 1200 s rose to 416.93 nM at 1800 s), and the neuron morphology, including axonal changes, showed significant alterations. One primary-cultured hippocampal neuron cell ruptured and died at 1900 s.

**Fig. 8:**
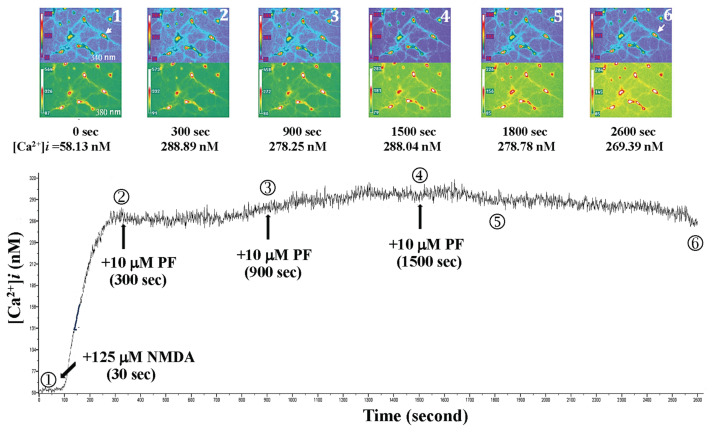
Effects of 3 repeated doses of 10 μM PF on NMDA-induced calcium influx and neuron morphology in primary cultured hippocampal neurons. NMDA was added to the primary-cultured hippocampal neurons 30 s after the beginning of fluorescent exposure; the Ca^2+^ level rose from 58.13 nM (0 s) to 288.89 nM (300 s). Three doses of 10 μM PF were administered at 300 s, 900 s, and 1500 s, respectively. The neuron morphology remained normal, and the neurons survived.

When 125 μM NMDA was added to the primary-cultured hippocampal neurons at 30 s after the beginning of fluorescent exposure, NMDA-mediated Ca^2+^ influx and the Ca^2+^ level rose from 84.05 nM (0 s) to 433.74 nM (200 s), as shown in Fig. 7. 10 μM PF was added at 300 s, at this point, the Ca^2+^ level decreased to 417.05 nM. The primary-cultured hippocampal neurons exhibited normal morphology. However, after prolonged fluorescence exposure (at 1200 s), the intracellular Ca^2+^ level in the primary-cultured hippocampal neurons began to rise (253.66 nM at 1200 s rose to 416.93 nM at 1800 s), and the neuron morphology, including axonal changes, showed significant alterations. One primary-cultured hippocampal neuron ruptured and died at 1900 s. Moreover, three doses of 10 μM PF were administered to the primary-cultured hippocampal neurons at 300 s, 900 s, and 1500 s, respectively. The neuron morphology, including axonal changes, was observed, and the neurons survived as indicated in Fig. 8.

**Fig. 9:**
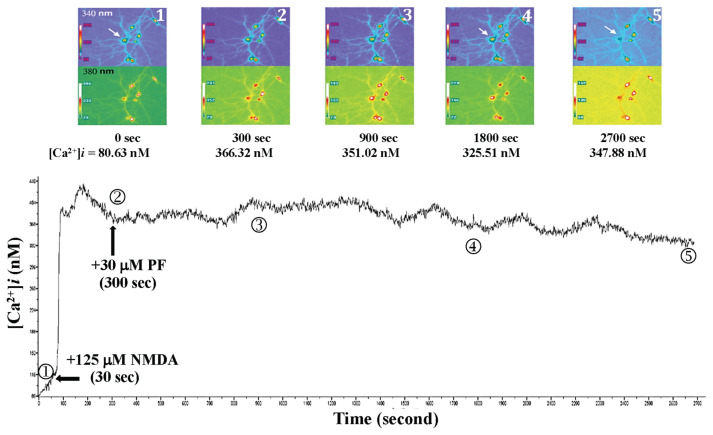
Effects of 30 μM PF on NMDA-induced calcium influx and neuronal morphology in primary cultured hippocampal neurons. NMDA was added at 30 s after the beginning of fluorescent exposure, and the Ca^2+^ level rose from 80.63 nM (0 s) to 366.32 nM (300 s). PF was added at 300 s, and the intracellular Ca^2+^ level fluctuated between 330 and 360 nM. The neuronal morphology was observed between 1600 and 1800 s; one primary-cultured hippocampal neuron ruptured and died at 2700 s.

When 125 μM NMDA was added to the primary-cultured hippocampal neurons at 30 s after the beginning of fluorescent exposure, NMDA-mediated Ca^2+^ influx and the Ca^2+^ level rose from 80.63 nM (0 s) to 366.32 nM (300 s), as shown in Fig. 9. 30 μM PF was added at 300 s. The intracellular Ca^2+^ level in the primary-cultured hippocampal neurons fluctuated between 330 and 360 nM. Moreover, the neuron morphology, including axonal changes, was observed between 1600 and 1800 s; one primary-cultured hippocampal neuron cell ruptured and died at 2700 s, and other neurons remained survived.

**Fig. 10:**
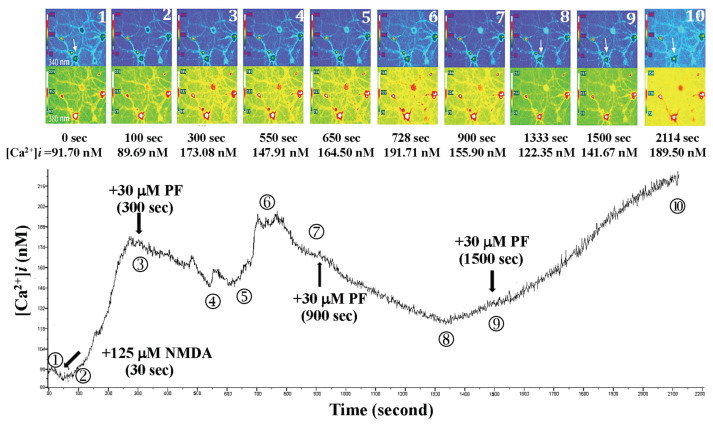
Effects of 3 repeated doses of 30 μM PF on NMDA-induced calcium influx and neuron morphology in primary cultured hippocampal neurons. NMDA was added 30 s after the beginning of fluorescent exposure, the Ca^2+^ level rose from 91.70 nM (0 s) to 173.08 nM (300 s). When 3 repeated doses of 30 μM PF were added at 300, 900, and 1500 s, respectively, the intracellular Ca^2+^ level fluctuated between 122.35 and 189.50 nM. The morphological structures (such as axons or dendrites) of primary-cultured hippocampal neurons remained intact, and no cell death was observed.

As shown in Fig. 10, 125 μM NMDA was added 30 s after the beginning of fluorescent exposure, the Ca^2+^ level rose from 91.70 nM (0 s) to 173.08 nM (300 s). When 30 μM PF was added three times at 300, 900, and 1500 s, respectively, as shown in Fig. 10. Intracellular Ca^2+^ levels in the primary-cultured hippocampal neurons fluctuated between 122.35 and 189.50 nM, and the morphological structures (such as axons or dendrites) of primary-cultured hippocampal neurons remained intact, and no cell death was observed.

**Fig. 11:**
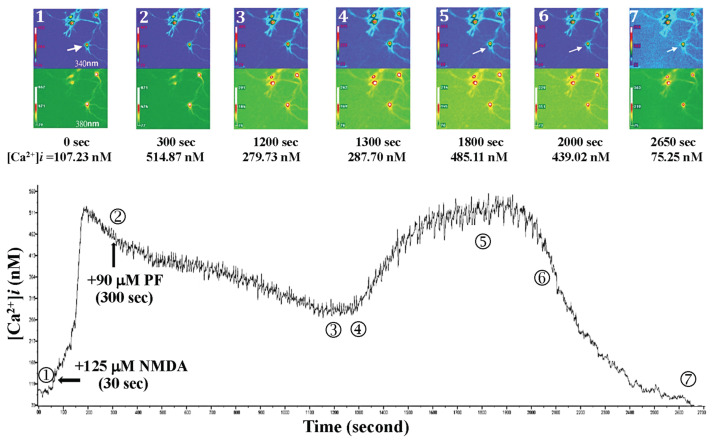
Effects of 90 μM PF on NMDA-induced calcium influx and neuron morphology in primary cultured hippocampal neurons. NMDA was added to the primary-cultured hippocampal neurons at 30 s after the beginning of fluorescent exposure, and the intracellular Ca^2+^ level increased from 107.23 nM (0 s) to 514.87 nM (300 s). PF was added at 300 s; the Ca^2+^ level was decreased to 279.73 nM (at 1200 s). After prolonged fluorescence exposure, the Ca^2+^ level began to increase to 485.11 nM (at 1800 s). At this moment, the neuron’s morphology changed, but it survived, and the Ca^2+^ level was 75.25 nM (at 2650 s). The above results indicate that a single application with a high dose of PF (90 μM) protected hippocampal neurons from NMDA-mediated calcium influx-induced neuronal death.

As shown in Fig. 11, 125 μM NMDA was added to the primary-cultured hippocampal neurons at 30 s after the beginning of fluorescent exposure, and the intracellular Ca^2+^ level increased from 107.23 nM (0 s) to 514.87 nM (300 s). After 90 μM PF was added at 300 s, the Ca^2+^ level was decreased to 279.73 nM (at 1200 s); however, after prolonged fluorescence exposure, the Ca^2+^ level began to be elevated to 485.11 nM (at 1800 s). At this moment, the neuron’s morphology changed, but it survived, and the Ca^2+^ level was 75.25 (at 2650 s). The above results indicate that a single application with a high dose of PF (90 μM) protected hippocampal neurons from NMDA-mediated calcium influx-induced neuronal death.

### 3.6. Effects of calcium channel modulators and/or PF on NMDA-induced calcium influx and neuron morphology in primary-cultured hippocampal neurons

#### 3.6.1. Effects of ruthenium red (RuR) or combined with PF on NMDA-induced calcium influx and neuron morphology in primary-cultured hippocampal neurons

**Fig. 12:**
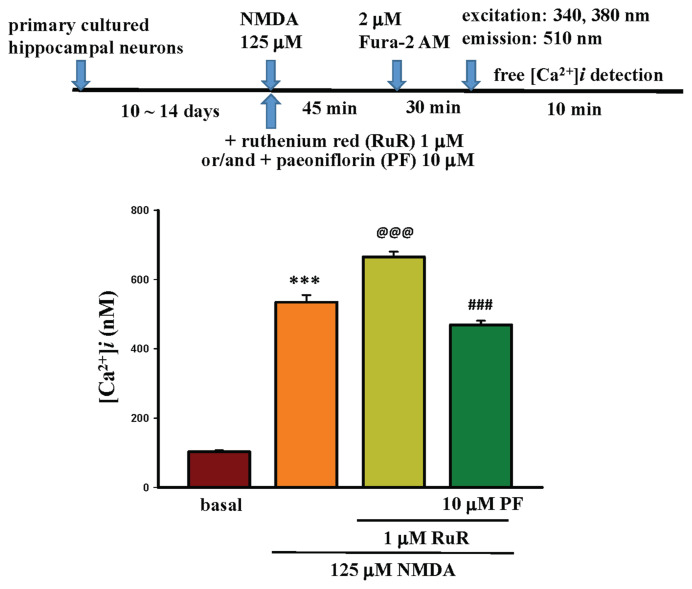
Effects of 1 μM Ruthenium Red (RuR) and/or 10 μM PF on 125 μM NMDA-induced calcium influx in primary-cultured hippocampal neurons. The procedure flow chart is shown in the upper panel of Fig. 12. NMDA co-treated with RuR for 45 min, the intracellular calcium level was 665.68 nM, as shown in the lower panel (P < 0.001, compared with the NMDA-treated group, 534.58 nM). When PF was added alongside RuR, the intracellular calcium level was decreased from 665.68 nM to 469.05 nM, resulting in a decrease of 29.54 %, as shown in the lower panel. The above data indicate that RuR inhibited the process of intracellular calcium recycling into the endoplasmic reticulum (ER), and PF reversed this effect (P < 0.001, compared with the NMDA + RuR group).

After adding 125 μM NMDA and 1 μM RuR to primary-cultured hippocampal neurons for 45 min, the intracellular calcium level in primary-cultured hippocampal neurons was 665.68 nM, as shown in the lower panel of Fig. 12, representing a significant increase compared to the calcium level in NMDA-treated neurons-treated hippocampal neurons (534.58 nM, P < 0.001). Significant calcium level increase was observed following the addition of RuR (24.5 %). Furthermore, when PF was added alongside RuR, the intracellular calcium level from 665.68 nM decreased to 469.05 nM, resulting in a decrease of 29.54 %, as shown in the lower panel of Fig. 12. The results indicate that PF reverses the NMDA + RuR-increased intracellular calcium in neuronal cells (P < 0.001).

#### 3.6.2. Effects of ω-conotoxin MVIIC (ω-Cono) or combined with PF and/or with RuR on NMDA-induced calcium influx and neuron morphology in primary-cultured hippocampal neurons

**Fig. 13:**
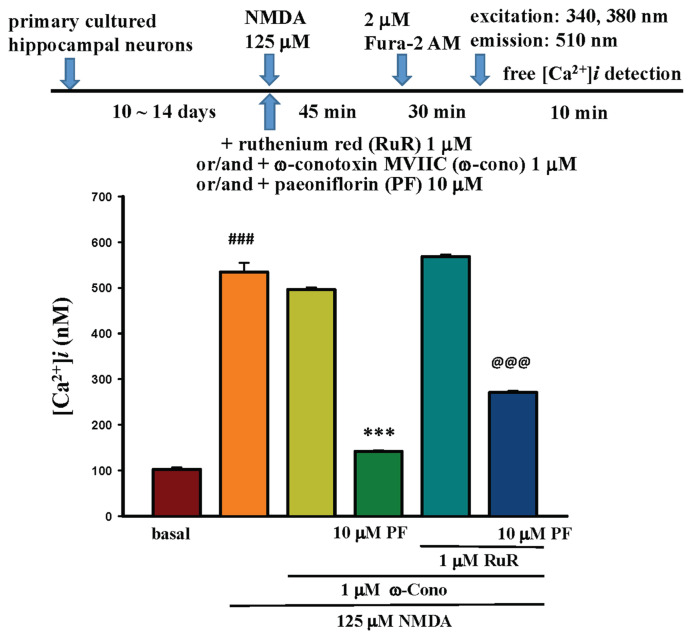
Effects of 1 μM ω-Conotoxin MVIIC (ω-Cono) or combined with 10 μM PF and/or with 1 μM RuR on NMDA-induced calcium influx in primary-cultured hippocampal neurons. The procedure flow chart is shown in the upper panel of Fig. 13. Incubating primary-cultured hippocampal neurons with 125 μM NMDA and 1 μM ω-Cono for 45 min, the intracellular calcium level was 496.29 nM. While adding NMDA, ω-Cono, and RuR to primary-cultured hippocampal neurons, the intracellular calcium level was 568.51 nM. Moreover, incubating with NMDA, ω-Conotoxin MVIIC, Ruthenium Red (RuR), and 10 μM PF for 45 min, the intracellular calcium level was 270.94 nM, as shown in the lower panel. The basal calcium level was 102.67 nM, in NMDA-incubation for 45 min was 534.58 nM, 496.29 nM in NMDA + ω-Cono group, and 141.51 nM in NMDA + ω-Cono + PF group; however, the calcium level in NMDA + ω-Cono + RuR group was 568.51 nM, and 270.93 nM in NMDA + ω-Cono + RuR + PF group. PF significantly decreased the intracellular calcium level induced by NMDA and NMDA co-treated with ω-Cono or RuR (P < 0.001).

*ω-Cono* is a N-type calcium channel inhibitor [47]. After incubating primary-cultured hippocampal neurons with 125 μM NMDA and 1 μM ω-Cono for 45 min, the intracellular calcium level in primary-cultured hippocampal neurons was 496.29 nM. While adding 125 μM NMDA, 1 μM ω-Cono, and 1 μM RuR to primary-cultured hippocampal neurons, the intracellular calcium level was 568.51 nM. Moreover, incubating with 125 μM NMDA, 1 μM ω-Conotoxin MVIIC, 1 μM Ruthenium Red (RuR), and 10 μM PF for 45 min, intracellular calcium level was 270.94 nM, as shown in the lower panel of Fig. 13. The basal calcium level was 102.67 nM, in 125 μM NMDA-incubated for 45 min was 534.58 nM, 496.29 nM in 125 μM NMDA + 1 μM ω-Cono group, and 141.51 nM in 125 μM NMDA + 1 μM ω-Cono + 10 μM PF group; however, the calcium level in 125 μM NMDA + 1 μM ω-Cono + 1 μM RuR group was 568.51 nM, and 270.93 μM in 125 μM NMDA + 1 μM ω-Cono +1 μM RuR + 10 μM PF group. In the presence of 10 μM PF, NMDA, and calcium channel modulators, the intracellular calcium level was significantly decreased (P < 0.001).

### 3.7. In silico molecular docking analysis of the PF with NMDA receptor complex

**Fig. 14:**
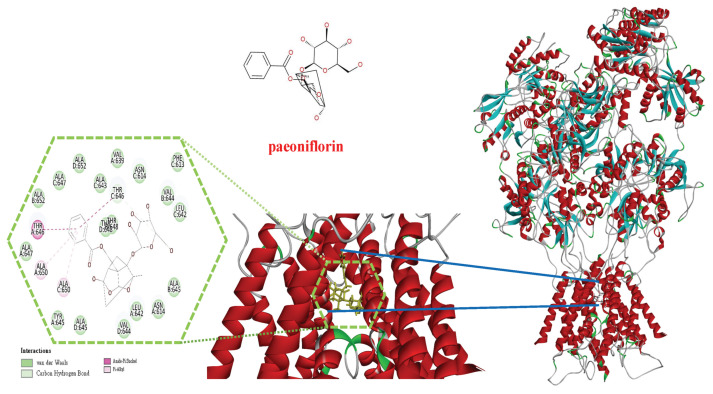
In silico molecular docking analysis of the PF with the NMDA receptor complex. The results of molecular docking analysis demonstrated that PF occupied the binding site of the NMDA receptor. PF formed multiple interactions with residues PHE613 (chain C), ASN614 (chain A and C), VAL639 (chain A), LEU642 (chain A and C), ALA643 (chain C), VAL644 (chain B and D), TYR645 (chain A), ALA645 (chain C), THR646 (chain A and C), ALA647(chain C), THR648 (chain D), ALA650 (chain A and C), ALA652 (chain B and D) of NMDA receptor. The binding energy of PF with the NMDA receptor is −48.5188 kcal/mol.

We conducted molecular docking analysis of PF with the NMDA receptor. The results of molecular docking analysis demonstrated that PF occupied the binding site of NMDA receptor (Fig. 14). The PF formed multiple bond interaction with residues PHE613 (chain C), ASN614 (chain A and C), VAL639 (chain A), LEU642 (chain A and C), ALA643 (chain C), VAL644 (chain B and D), TYR645 (chain A), ALA645 (chain C), THR646 (chain A and C), ALA647 (chain C), THR648 (chain D), ALA650 (chain A and C), ALA652 (chain B and D) of NMDA receptor (Fig. 14). The binding energy of PF with the NMDA receptor is −48.5188 kcal/mol. Our results suggest that PF can bind to the NMDA receptor binding site and further interfere with the activity of the NMDA receptor.

## 4. Discussion

Conventional antidepressants such as TCAs, MAOIs, SSRIs, SNRIs, and ketamine are commonly used and have a relatively good therapeutic effect. However, these antidepressants also elicit adverse effects in some patients [48,49], such as SSRIs and SNRIs, which usually cause digestive, sexual, and sleeping disorders; TCAs can cause dry mouth, constipation, drowsiness, and weight gain [50]; ketamine causes psychotomimetic, cardiovascular, neurological or cognitive side-effects [51]. Therefore, the search for new antidepressants is an urgent task for scientists. Remarkably, no side effects such as ataxia, motor incoordination, memory impairment and psychomimetic effects were found in a previous report [28] and reveals the superiority of PF.

The hippocampus regulates stress response [6] and plays an important role in the regulation of mood. Exposure to stress leading to hippocampal neuron loss has been well-studied in animal studies [7–10]. NMDARs are present in both pyramidal neurons and interneurons of the hippocampus and play important roles in the etiopathology of specific psychiatric disorders [52]. Previous research reports indicated that the NMDAR is associated with specific central nervous system excitatory symptoms, such as epilepsy and psychosis [53]. When NMDARs are overactivated, it causes excessive neuronal excitation. It triggers psychosis and epileptic seizures, which is known as neuro-excitotoxicity, thus leading to hypoxia in neurons, further causing apoptosis and necrosis, ultimately resulting in permanent damage to neurons [54]. In patients with depression, it has been found that the function of the glycine binding site on NMDA receptors gradually diminishes, accompanied by a decrease in blood glycine concentration or an increase in the serine-to-glycine ratio [55]. However, some scholars have pointed out that this is related to an increase in serine concentration in plasma [56].

Additionally, some researchers have attempted to study the binding of antidepressant drugs to NMDARs in the brains of mice [57–60]. Furthermore, administration of antidepressants can alter NMDAR function. Long-term administration of antidepressants or electroconvulsive shock (ECS) reduces the strength and affinity of glycine displacement from [^3^H]5,7-diCl-KTN binding; similarly, the competitive NMDAR antagonist [^3^H] CGP-39653 also exhibits reduced affinity for glycine displacement binding [61–63]. The above reports indicate that the effects of antidepressants are related to the inhibition of NMDARs. Therefore, the importance of NMDAR modulators as therapeutic targets for relieving mood disorders and the glutamate/NMDAR signaling plays a promising role in the development of antidepressants is reported by several researchers [15,22–24].

PF demonstrates a wide range of pharmacological activities, including antioxidation, anti-inflammation, smooth muscle relaxation, analgesia, hepatic protection, and neuroprotection, among others. PF exhibits anti-inflammatory and neuroprotective effects in attenuating ischemia/reperfusion-induced brain damage via inhibiting NFκB, IL-1β, TNFα, and its protective effect is related to the modulation of the Ras/MEK/ERK signaling pathway [31]. PF also inhibits various condition-induced algesia, including formalin-induced algesia [31], and excitatory amino acid (EAA) agonistinduced nociception [28]. PF exhibits its analgesic effect against formalin-induced nociception via activating κ-opioid receptors. In contrast, PF inhibits EAA agonist-induced nociceptive behavior, which is mediated by NMDAR modulation, especially targeting the NR2B subunit [28]. Since NMDAR is one of the receptors that glutamate binds to, it has been widely discussed that impaired glutamatergic neurotransmission has been implicated in several neurological diseases, such as neuropathic pain [64], acute stroke [65], epilepsy [66], and depression [54]. The excitatory effect of glutamate is mediated via NMDAR [67]. Drug candidates targeting NMDAR for glutamate-related neurological disorders might provide a promising platform.

Previous reports indicated the glutaminergic system is also involved in the pathophysiology of depression [12,13], and NMDARs are involved in the pathophysiology of mental disorders, including depression [21,23,68–70]. Substances that block NMDARs could have potential clinical use in the management of depression. NMDAR antagonists, such as psychotomimetic effects, ataxia, motor incoordination, and memory impairment, cause numerous side effects. Developing new candidates that can inhibit excitation and maintain the normal function of NMDAR would be a better strategy for depression treatment. PF targeting NR2A and NR2B subunits was corroborated *in silico* experiments, and docking energy data revealed that PF had more vigorous binding activity in NR2A and NR2B of NMDAR, and the result of the docking procedure corresponded to the experiment of an animal model [28]. Co-administration of PF with antisense ODNs of NMDAR subunits in mice produced no side effects, such as ataxia, motor incoordination, memory impairment, and psychomimetic effects. This finding highlights the superiority of PF, which may act partially via the modulation of NMDAR [28].

NMDAR contains an ion channel allowing calcium influx; upon NMDAR activation, an increase in intracellular calcium levels will lead to excitotoxicity, further leading to neuronal damage and causing neurodegenerative diseases, including depression. In this present study, PF significantly decreased the NMDA-induced intracellular calcium level increase and inhibited calcium channel modulator-induced calcium level changes. RuR is a non-competitive inhibitor of the mitochondrial Ca^2+^ uniporter, which abolishes Ca^2+^ uptake and inhibits intracellular calcium release by acting as a blocker of ryanodine receptors (RyRs) and N-type channel, and able to enhance intracellular free Ca^2+^ [43]. In the presence of NMDA and RuR, the intracellular calcium level increased, as shown in Fig. 12, revealing that RuR abolished calcium uptake and increased intracellular calcium level induced by NMDA. However, after co-treatment with PF, PF significantly decreased the intracellular calcium level caused by NMDA and RuR cotreatment. The data indicate that PF reversed the RuR-increased intracellular calcium level. Another calcium channel modulator used in this study was ω-Cono. ω-Cono is a peptide toxin that inhibits N-type calcium channels [44] and decreases the calcium level in neurons. In the presence of NMDA and ω-Cono, the intracellular calcium level was slightly decreased (534.58 nM–496.29 nM), when PF was present, the intracellular calcium returning to near basal level (496.29 nM–141.51 nM) as shown in Fig. 13. Additionally, in the presence of NMDA, ω-Cono, and RuR, the intracellular calcium level increased to 568.50 nM, and reduced to 270.94 nM in the presence of PF. The above data indicated that PF can affect the effects of calcium channel modulators on the NMDA-induced calcium influx and maintain the intracellular calcium at a stable and non-toxic level. These data suggest that the neuroprotective effects of PF are related to its effect against NMDA-induced calcium overload, and were supported by a previous report that reported PF can promote neuronal viability and inhibit neuronal apoptosis via down-regulation of the Ca^2+^/CaMKIL/CREB signaling pathway in a cerebral ischemia injury model [71]. In that published paper, the doses of PF (100 μM and 200 μM) used are 10–20 times higher than those used in this study (10 μM) and in previous papers published by our group [31,72].

Besides the involvement of glutamate in mental disorders, there is a strong association between reduced levels of serotonin metabolite, 5-hydroxyindoleacetic acid (5-HIAA) in the cerebrospinal fluid (CSF) and an increased tendency toward suicidal behavior, further implicating serotonergic dysfunction in the severity and outcome of depressive disorders [73]. Cumulative reports have proved the potential effects of phytoconstituents, including PF, in the treatment of depression that the phytoconstituents mediate their antidepressive effect through upregulation of monoaminergic neurotransmitters level, inhibition of MAO activity, and downregulation of oxidative stress and neuroinflammation [74]. Biochemical analysis revealed that PF treatment significantly increased the level of 5-HIAA and 5-HT in the hippocampus region [35,75]. Moreover, plasma and hippocampus monoamine analysis showed that PF treatment significantly increased the levels of dopamine, norepinephrine, and serotonin [35] and exhibits antidepressant effects. PF preserves serotonergic and dopaminergic neurotransmission and suppresses glutamate-induced excitotoxicity by regulating synaptic proteins engaged in glutamate release, which might contribute to the relief of depression symptoms.

A previous report indicates that PF exerts its antidepressant effects by inhibiting glutamate release and is related to the suppression of SNARE complex proteins [39]. Additionally, it modulates the glutamate receptors and transporters in the hippocampus, leading to a significant reduction in the expression of the NR1 and NR2 subunits. These findings confirm the previous report that the neuroprotection of PF via the modulation of NMDAR, docking energy data revealed that PF had more vigorous binding activity in NR2A and NR2B than NR2C of NMDA receptors, especially the NR2B subunit [28].

To confirm the effect of PF on NMDAR through its binding to the NMDAR, an *in silico* molecular docking analysis of PF with the NMDAR complex was performed. The results of molecular docking analysis demonstrated that PF occupied the binding site of the NMDAR, as shown in Fig. 14. The PF formed multiple bond interactions with residues in chains A, B, C, and D of the NMDAR, and with a binding energy of −48.5188 kcal/mol. Our results suggest that PF can bind to the NMDA receptor binding site and further interfere with the activity of the NMDA receptor.

## 5. Conclusions

**Fig. 15:**
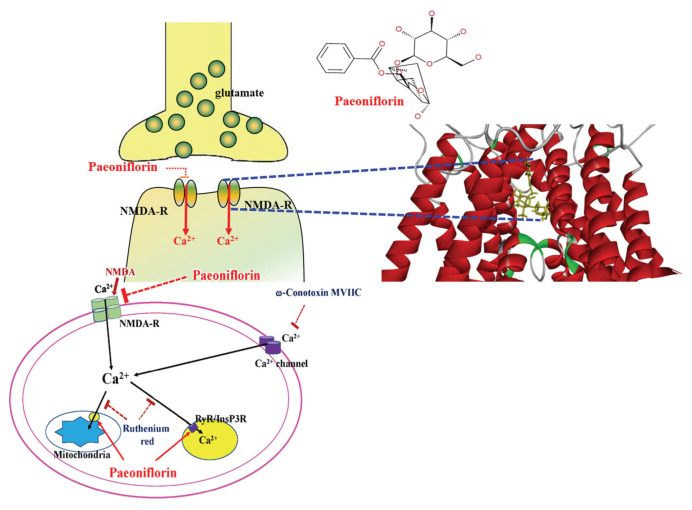
Proposed action mechanism of paeoniflorin (PF) on NMDA-induced calcium influx in primary-cultured hippocampal neurons. PF binds to NMDA receptor binding sites and decreases intracellular calcium by attenuating ω-Conotoxin MVIIC and reversing Ruthenium Red-induced calcium level increase. PF binds to the NMDA receptor and acts as an NMDA receptor modulator, leading to a decrease in the intracellular calcium level and exhibiting neuroprotection.

PF, a phytoconstituent from *Paeonia lactiflora* Pallas, is more broadly available, acceptable, and has fewer side effects as compared to conventional antidepressants and provides a wide range in the treatment of depression. PF binds to the NMDA receptor to attenuate NMDA-mediated EPSPs and calcium influx, thereby exhibiting neuroprotection and contributes to its potential as an antidepressant. However, clinical findings are needed to ensure the safety and therapeutic efficacy of PF as an antidepressant. The proposed mechanism of action of paeoniflorin (PF) binds to the NMDA receptor and decreasing NMDA-induced intracellular calcium increase, as shows in Fig. 15.
